# Intussusception in Cronkhite–Canada Syndrome

**DOI:** 10.3390/diagnostics16152415

**Published:** 2026-07-31

**Authors:** Li Ma, Ji Li, Xiaoyin Bai, Qingli Zhu

**Affiliations:** 1 Department of Ultrasound, Peking Union Medical College Hospital, Beijing 100730, China; litangpai@163.com; 2 Department of Gastroenterology, Peking Union Medical College Hospital, Beijing 100730, China; liji0235@pumch.cn (J.L.); baixiaoyin@pumch.cn (X.B.)

**Keywords:** Cronkhite–Canada syndrome, intussusception, cross-sectional imaging

## Abstract

Cronkhite–Canada syndrome is a rare, non-hereditary polyposis syndrome characterized by non-specific gastrointestinal symptoms accompanied by alopecia, cutaneous hyperpigmentation, and nail dystrophy. Characteristic endoscopic findings are diffuse sessile polypoid lesions with edematous mucosa. We report a case of a 44-year-old woman in whom intestinal ultrasound and CT revealed diffuse mucosal thickening and an ileocecal intussusception—findings that may not be pathognomonic but are highly unusual in adults. These imaging features served as critical red flags that directed the clinical suspicion toward Cronkhite–Canada syndrome and prompted timely endoscopic confirmation.

**Figure 1:**
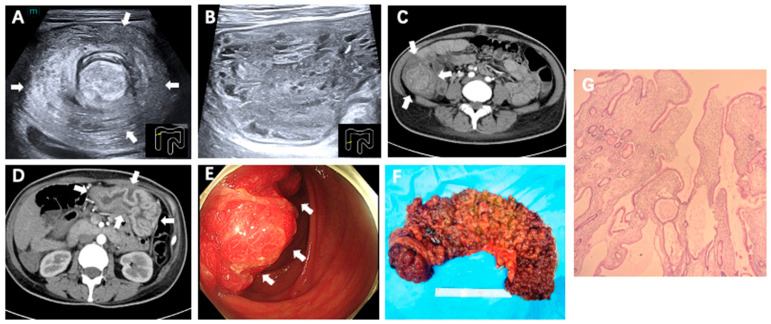
A 44-year-old woman was admitted to our hospital due to continuous abdominal discomfort and diarrhea for over one year. The patient also complained of hypogeusia, alopecia, nail deformation, and palm hyperpigmentation. The patient was given a short course of oral corticosteroids 10 months ago, and her symptoms have since been partially relieved. Main laboratory test results: Body Mass Index, 21.1 kg/m^2^; white-blood-cell count, 8.27 × 10^9^/L; hemoglobin, 108 g/L; albumin, 2.3 g/dL; C-reactive protein, 9.44 mg/L; and mild urine protein, (1+). Intestinal ultrasound revealed the concentric sign, indicating the intussusception of the terminal ileum into the ascending colon ((**A**), white arrows), and prominent mucosal thickening with small cystic spaces throughout the gastrointestinal tract, most notably in the ascending colon (**B**). Computed tomography (CT) also presented diffuse mucosal thickening and bowel-within-bowel configuration in the ileocecal region ((**C**), white arrows) and coarse mucosal folds of the stomach ((**D**), white arrows). Upper and lower endoscopy showed diffuse endoscopic polyposis extending from the stomach to the colon, most notably in the ascending colon ((**E**), white arrows). The patient was given 40 mg/day of prednisone after admission, but her symptoms were not fully relieved. The gastroenterologist successfully reduced the intussusception via colonoscopy; however, the patient subsequently experienced a recurrence complicated by acute intestinal obstruction, ultimately necessitating a right colectomy. Gross surgical specimen showed dense polypoid lesions (**F**). Histopathology (H&E, original magnification 40×) found these polypoid lesions were hamartomatous polyps featuring crypt architectural distortion and stromal edema without dysplasia (**G**). The constellation of clinical, endoscopic and histopathological features was consistent with Cronkhite–Canada syndrome (CCS), a non-familial polyposis syndrome characterized by non-specific gastrointestinal symptoms in association with alopecia, cutaneous hyperpigmentation, and nail dystrophy [1]. The diagnosis relies on characteristic endoscopic, dermatologic, and pathological features [2]. The pathological mucosal changes in CCS may cause protein leakage and lead to protein-losing enteropathy. Glucocorticoids are generally effective in CCS, and anti-tumor necrosis factor has been reported to be effective in refractory cases [3,4]. Importantly, a significant risk of malignant transformation persists; therefore, a rigorous, lifelong endoscopic surveillance strategy is essential [5,6]. Here, we report the imaging features of CCS as diffuse mucosal thickening and ileocecal intussusception. Several cases of ileocecal intussusception in CCS have been reported, but its incidence remains unclear [7,8]. Although these findings may not be pathognomonic, they can serve as critical red flags that direct clinical suspicion toward CCS and prompt timely endoscopic confirmation, thereby facilitating early diagnosis and appropriate management.

## Data Availability

The original contributions presented in this study are included in the article. Further inquiries can be directed to the corresponding author.

## Abbreviations

The following abbreviations are used in this manuscript:

CCS	Cronkhite–Canada syndrome
CT	Computed Tomography
